# Tri-State Dental Meeting

**Published:** 1894-10

**Authors:** 


### EDITORIAL.

### Tri-State Dental Meeting.

At the last annual meeting of the Ohio State Dental Society a resolution was offered proposing a joint meeting of the Ohio, Michigan and Indiana State Dental Societies to be held at a time and place to be fixed.

A committee of three was appointed to confer with similar committees that might be appointed by Michigan and Indiana. At the last annual meeting of these two States the subject was presented and the committees as contemplated were appointed. After some conference, by correspondence, these committees designated Old Point Comfort, and the 6th of August, as the place and time for their meeting. At this time a majority of each committee was present.

They were called together and took into consideration the question of holding such a meeting, after which it was unanimously decided that it was not only feasible but very desirable that such a meeting be held. The time fixed for it was the third Tuesday in June, 1895, the place selected was Detroit, Mich.

A committee of one from each State was appointed to constitute an executive committee. Dr. L. L. Barber, of Toledo, was made chairman of that committee; the other members were Dr. J. Ward House, of Grand Rapids, Mich., and Dr. G. E. Hunt, of Indianapolis, Indiana. A local committee was appointed to make arrangements for the accommodation of the meeting; Dr. George L. Field, of Detroit, was made chairman of that committee.

From the interest manifested by all those to whom this matter has been presented, it is very certain that a large and interesting meeting will be held.

A report of the progress of the various committees preparatory to this meeting will be given in the pages of the REGISTER from time to time. In the meantime communications in reference to it may be sent to Dr. L. L. Barber, of Toledo, or Dr. George L. Field, of Detroit.

We trust that there will be not less than one hundred members from each State, and two or three times that number if possible.

